# Hierarchically Porous Mullite Ceramics Assembled from Ultrathin Nanosheets for High-Temperature Thermal Insulation

**DOI:** 10.3390/ma19163433

**Published:** 2026-08-13

**Authors:** Zhongyan Wang, Anran Guo, Xueying Zhang, Jiaomei Ma, Jiachen Liu

**Affiliations:** 1 Key Laboratory of Advanced Ceramics and Machining Technology of Ministry of Education, School of Materials Science and Engineering, Tianjin University, Tianjin 300072, China; 18131126553@163.com (Z.W.); zxy_92@163.com (X.Z.); 2 Tianjin Cement Industry Design & Research Institute Co., Ltd., Tianjin 300131, China; majiaomei@sinoma-tianjin.cn

**Keywords:** mullite porous ceramics, ultrathin nanosheet, hierarchical structure, mechanical strength, thermal insulation

## Abstract

**Highlights:**

**What are the main findings?**

- Ultrathin mullite nanosheets (~2–3 nm thick) were synthesized via a chemical blowing method.
- Hierarchically porous ceramics achieved 90.75% porosity and a compressive strength of 0.38 MPa.
- Exhibited low apparent thermal conductivity (0.0756 W/(m·K)) and excellent stability up to 1500 °C.

**What are the implications of the main findings?**

- This work achieves a favorable balance between structural integrity and thermal insulation in highly porous ceramics.
- The nanosheet assembly strategy offers a new route for lightweight insulation materials in extreme environments.
- The approach may be extended to other ceramic systems for high-temperature thermal protection applications.

**Abstract:**

Mullite porous ceramics show exceptional promise for high-temperature insulation applications. However, the mechanical strength and thermal insulation performance of porous ceramics typically cannot be optimized simultaneously. Herein, we proposed a novel method to overcome this limitation by synthesizing hierarchically porous mullite ceramics assembled from ultrathin two-dimensional nanosheets. A chemical blowing method first synthesized ultrathin mullite nanosheets approximately 2–3 nm thick, which were subsequently assembled into a rigid hierarchical network by gel casting and freeze-drying. The influence of solid content and sintering temperature on the phase composition, microstructure, mechanical properties, and high-temperature thermal stability of porous ceramics was systematically investigated. The results indicate that the 20 wt.% sample sintered at 1200 °C exhibited the best performance. This optimal sample achieved a porosity of 90.75%, a compressive strength of 0.38 MPa, and excellent thermal insulation properties, including a low apparent thermal conductivity of 0.0756 W/(m·K) at room temperature and a back surface temperature of 253.2 °C after exposure to a 1200 °C flame. Crucially, this porous ceramic maintained structural stability up to 1500 °C. This nanosheet assembly strategy successfully reinforced the structural skeleton while inhibiting heat transfer. This strategy has great promise for the fabrication of lightweight porous ceramics designed for extreme environments.

## 1. Introduction

Extreme working environments in aerospace and energy industries call for lightweight thermal insulation materials with ever more demanding performance requirements [1,2,3,4]. Among numerous candidate materials, mullite porous ceramics are considered as an ideal thermal insulation material for extremely high-temperature environments due to their inherent low thermal conductivity [5,6], excellent high-temperature creep resistance [7,8,9], and outstanding chemical stability [10,11]. However, current research on porous mullite ceramics primarily focuses on materials composed of traditional micron and submicron particles [12,13,14], as well as micron and nanofibers [15,16]. For porous ceramics composed of particles, the increased contact area between particles during sintering intensifies solid-state heat transfer, thereby reducing thermal insulation performance [17,18]. To address this issue, high-aspect-ratio fibers have been introduced to construct an interconnected network structure that effectively reduces solid-phase heat conduction pathways [19,20]. However, the inherently weak bonding between the fibers results in a significant reduction in mechanical strength [21,22]. Consequently, this strategy effectively mitigates the trade-off between thermal insulation and mechanical integrity in highly porous ceramics [23,24,25].

To overcome the limitations of micro-sized particles and fibers, the use of ultrathin nanosheets as novel structural units has emerged as a highly promising strategy [26]. Their unique planar geometry allows ultrathin nanosheets to form a stable interlocking framework through extensive overlapping, endowing the material with excellent mechanical properties. Meanwhile, the nanosheets divide the internal space into isolated micropores, lengthening the heat conduction path and providing a large number of interfaces for phonon scattering. As a result, the material achieves exceptional thermal insulation without compromising its high strength [27,28].

Based on the above advantages, various porous ceramics synthesized using nanosheets have been reported [29,30]. For instance, Ji et al. [31] used two-dimensional α-Al_2_O_3_ nanosheets to assemble biphasic aerogels, thereby simultaneously optimizing mechanical strength and thermal insulation performance. This material has a compressive strength of 0.6 MPa at room temperature and a thermal conductivity of 0.029 W/(m·K). Xu et al. [32] constructed a three-dimensional structure from hexagonal boron nitride (h-BN) nanosheets, yielding a porous ceramic with exceptional structural stability. This material exhibited exceptional mechanical toughness (up to 95% superelasticity) and a minimized thermal conductivity of merely 0.020 W/(m·K).

Although successfully used to fabricate advanced porous structures in various material systems, nanosheets have not yet been demonstrated for application in mullite porous ceramics. This is mainly attributed to the difficulty in synthesizing mullite nanosheets with controllable sizes. Due to the inherent crystal growth characteristics of mullite, it tends thermodynamically to grow anisotropically along specific axes [33]. Consequently, conventional methods are primarily limited to producing one-dimensional fibers or zero-dimensional particles; isolating two-dimensional sheet-like structures has remained a significant challenge [34]. Furthermore, even if two-dimensional nanosheets are successfully prepared, their inherent high surface energy often leads to stacking and agglomeration, making it difficult for the nanosheets to be uniformly dispersed during the formation of a porous structure [35,36].

To overcome this inherent limitation, this study proposes a novel method that combines chemical blowing with gel-casting to fabricate hierarchically porous mullite ceramics. Initially, the chemical blowing method utilizes the in-situ release of gas to form a foaming agent during pyrolysis [37,38,39,40]. This gas generation causes the polymer gel precursor to bubble and burst, thereby effectively producing ultrathin mullite nanosheets. Subsequently, the synthesized nanosheets were assembled into an interconnected network structure using a gel-casting process. During the sintering process, the silica sol acted as an inorganic binder, effectively bridging the overlapping nodes of the nanosheets to create a structure with both an interlocking framework and a hierarchical porous structure. This work aimed to construct highly porous mullite ceramics with low thermal conductivity while maintaining the structural integrity of the nanosheet framework and high-temperature stability. Through this strategy, the nanosheets were assembled into a hierarchical porous network, providing an effective route to balance thermal insulation and mechanical integrity in highly porous mullite ceramics.

## 2. Materials and Methods

### 2.1. Materials

The blowing process was achieved by combining anhydrous glucose (C_6_H_12_O_6_, AR) as a carbon template and ammonium chloride (NH_4_Cl, AR) as a blowing agent. C_6_H_12_O_6_ and NH_4_Cl come from Tianjin Kemei Chemical Reagent Co., Ltd. (Tianjin, China). The as-prepared silica sol had a solid content of 40 wt.% and was diluted to 1 wt.% before being used as an inorganic binder for the mullite porous ceramics. This was prepared from tetraethyl orthosilicate (TEOS, Si(OC_2_H_5_)_4_, AR), deionized water, and ethanol (ETOH, C_2_H_6_O, AR). Agarose (C_24_H_38_O_19_, AR) was used as the gelling agent for the mullite porous ceramics. Tetraethyl silicate, alcohol and agarose all come from Shanghai Maclin Biochemical Technology Co., Ltd. (Shanghai, China).

### 2.2. Preparation of Ultrathin Mullite Nanosheets

**Figure 1:**
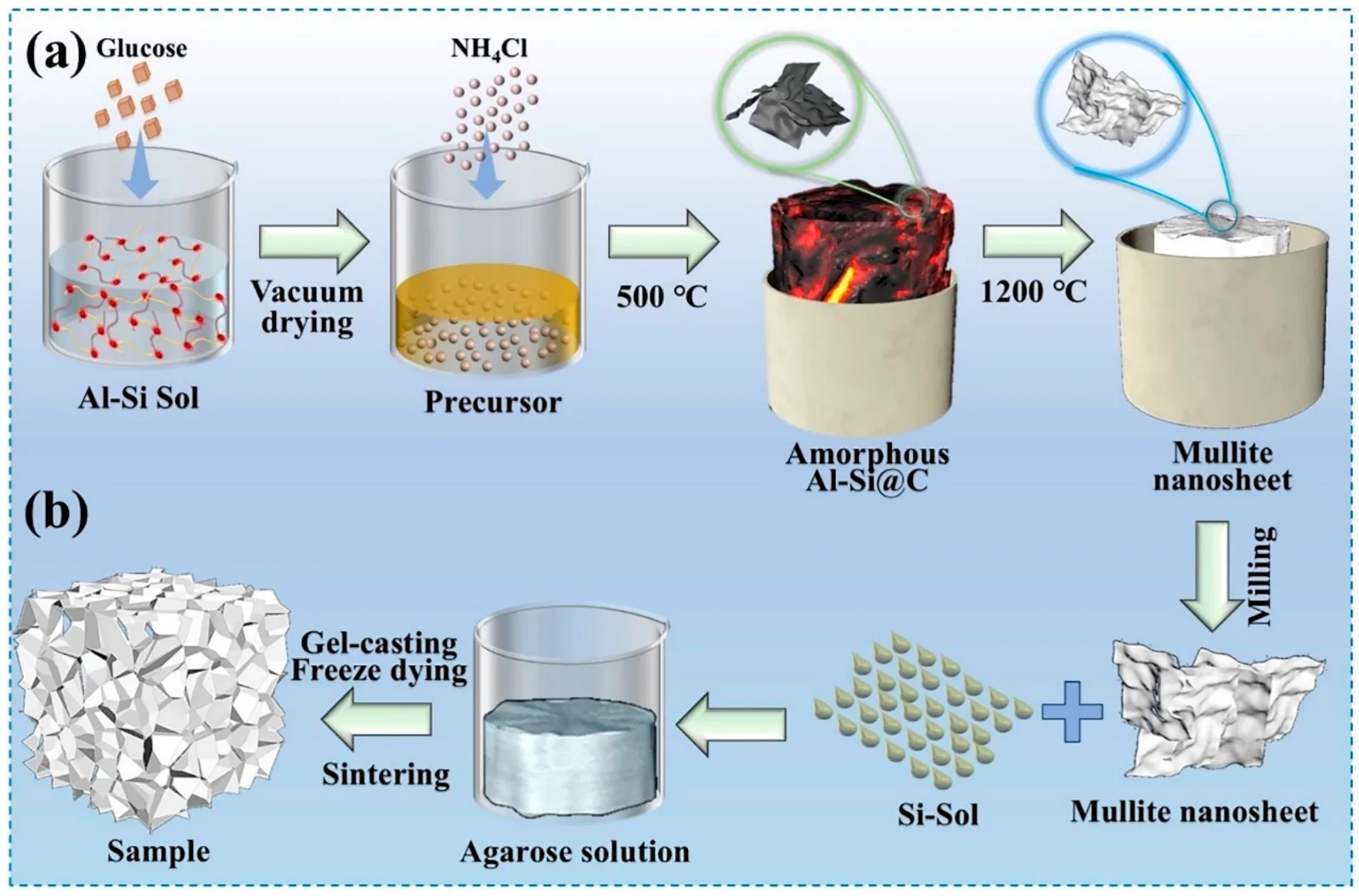
(**a**) Preparation of mullite nanosheets and (**b**) the production route for mullite porous ceramics.

The process for preparing ultrathin mullite nanosheets through chemical blowing is shown in Figure 1a. The aluminum silicate sol used as the precursor for mullite was prepared according to the method described in our previous work [41,42]. To synthesize the precursor, 1 mol of anhydrous glucose was first added to the aluminum silicate sol and stirred at 40 °C for 30 min until a clear, viscous mixture was achieved. Following a 48 h vacuum drying process at 70 °C, the mixture evolved into a thick, clay-brown, highly viscous state. Subsequently, the uniform precursor was obtained by thoroughly incorporating 1 mol of NH_4_Cl into this dense mixture. Heating a small amount of the precursor at 500 °C for 10 min in a high-temperature furnace produced a fluffy black product, which was named amorphous Al–Si@C. To obtain ultrathin mullite nanosheets, the amorphous Al–Si@C was finally sintered in air at 1200 °C with a heating rate of 4 °C/min for 1 h.

### 2.3. Preparation of Mullite Porous Ceramics

As shown in Figure 1b, the porous mullite ceramics were typically fabricated by the following process. First, a 2 wt.% solution was prepared by dissolving 0.16 g of agarose in 8 mL of deionized water. The solution was heated to 95 °C and stirred continuously for 10 min. During this process, the agarose was completely dissolved in the hot water. Then, the mullite nanosheet powder was thoroughly mixed with the agarose solution to prepare mixtures with solid contents of 10 wt.%, 15 wt.%, 20 wt.%, and 25 wt.%. Finally, 8 mL of 1 wt.% silica sol was added to the mixture, which was then stirred evenly and poured into a 2 cm × 2 cm × 2 cm mold. After curing, the mold was removed to obtain the preform. The agarose gel network was used to maintain the homogeneous distribution and structural integrity of the nanosheets during freezing. The obtained powder was frozen in a -80 °C refrigerator for 12 h, and then freeze–dried in a freeze-drying machine (FD-1A-50, Beijing, China) for 48 h. During freeze-drying, ice crystals served as a temporary template to generate interconnected pore channels, while the stacked nanosheets contributed to the formation of secondary pores within the pore walls. (Supporting Information, Figure S5b) Finally, the sample was heated in air at a rate of 4 °C /min to 1200 °C and held for 2 h. During sintering, the decomposition of agarose promoted the opening of pores, while silica-sol-derived bonding and solid-state diffusion between nanosheets stabilized the hierarchical porous framework. (Supporting Information, Figure S5c).

### 2.4. Characterization

Morphological observations of mullite porous ceramics were carried out using field emission scanning electron microscopy (SEM, Sigma 300, Zeiss, Germany), coupled with energy dispersive spectroscopy (EDS, Oxford, High Wycombe, UK) for simultaneous elemental mapping. X-ray diffraction (XRD) analysis was performed on the precursors and mullite porous ceramics at different temperatures using a D8 Advance diffractometer (Bruker, Germany), with a scan rate of 2°/min and a scanning range of 2θ range of 10–80° to determine the phase composition. The thermal evolution of the precursor and the porous ceramics was performed using a thermogravimetric-differential scanning calorimetry analyzer (TG-DSC, Netzsch STA 449 C, Selb, Germany) from room temperature up to 1500 °C in an air atmosphere. The chemical bonding characteristics were analyzed using a Fourier transform infrared (FTIR) spectroscopy (IR Prestige-21, Shimadzu, Japan) in the wavenumber range of 500–4000 cm^−1^. The thickness of the mullite nanosheets was determined using atomic force microscopy (AFM, Multimode 8, Bruker, Santa Barbara, CA, USA). Linear shrinkage was calculated from the sample dimensions before and after sintering. The bulk density of the sintered specimens was calculated from the dry mass and external geometric volume according to ρ = m/V, where m is the mass and V is the geometric volume of the sample. The apparent porosity was measured by the Archimedes method using deionized water as the immersion medium. The specimens were vacuum-immersed in deionized water for 30 min to saturate the accessible pores. The dry mass (m_0_), saturated mass in air (m_1_), and suspended mass in water (m_2_) were recorded, and the apparent porosity was calculated according to p = [(m_1_ − m_0_)/(m_1_ − m_2_)] × 100%. The pore size distribution and specific surface area of the porous ceramics (10 mm × 10 mm × 10 mm) were determined using mercury intrusion porosimetry (AutoPore IV 9500, Norcross, GA, USA). Compression strength was tested using a universal testing machine (CMT4304, MTS Systems Co., Ltd., Norcross, GA, USA) applied at 0.05 mm/min, with results averaged over five specimens. The thermal images of the samples were acquired using an infrared thermal imager (LT7-P, Zhejiang Dali Technology Co., Ltd., Hangzhou, China). The high-temperature measurement range of 100–600 °C was used during the flame test. Therefore, temperatures below 100 °C were outside the valid measurement range. As shown in Figure S2 (Supporting Information), the sample was horizontally placed on a fiber cloth support, and its front surface was continuously exposed to a butane flame. The temperature of the flame-facing surface exceeded 1200 °C during the test. The distance between the flame nozzle and the sample surface was fixed at 10 cm, while the backside temperature was monitored using an infrared thermal imaging camera positioned approximately 20 cm from the sample. The lateral surfaces were not additionally insulated. The same sample dimensions and experimental configuration were maintained for all tests. The room-temperature apparent thermal conductivity and thermal diffusivity of the porous ceramics were measured using a Hot Disk TPS 3500 analyzer (Sweden) based on the transient plane source method. Samples with dimensions of 10 mm × 10 mm × 5 mm were tested at approximately 25 °C under ambient conditions. The heating power and measurement time were adjusted to maintain a temperature rise of 2–5 K and a measurement-time/characteristic-time ratio of 0.33–1. The volumetric heat capacity was obtained simultaneously from the TPS analysis. Each condition was measured 3 times.

## 3. Results

### 3.1. Synthesis Mechanism and Characterization of Ultrathin Mullite Nanosheet

Ultrathin mullite nanosheets were synthesized via chemical blowing (Figure 1a) and investigated for the chemical reactions, phase transitions, and morphological evolution from the polymer precursor to the crystalline nanosheets.

**Figure 2:**
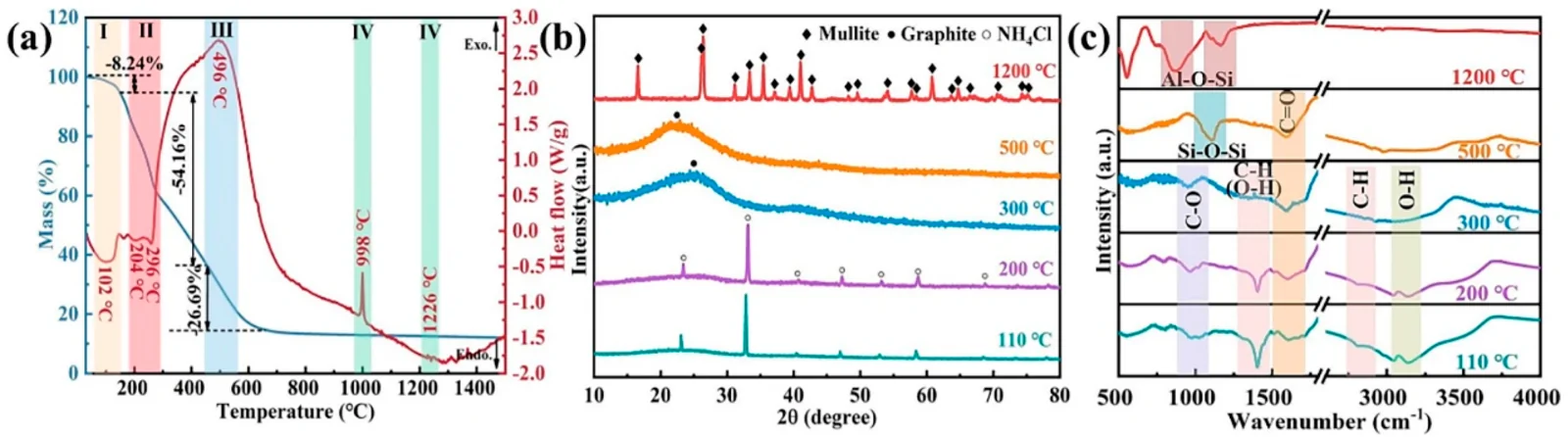
(**a**) TG-DSC curves of the precursor at 25–1500 °C under an air atmosphere, (**b**) XRD patterns of the precursors, (**c**) FTIR spectra of the precursors.

As shown in Figure 2a, the thermal behavior of the precursor was evaluated using TG-DSC under an air atmosphere from room temperature to 1500 °C. The weight loss of the precursors occurred in four stages. During the first stage, which occurred below 200 °C, the sample experienced a slight weight loss of around 8.39%. The primary cause was the evaporation of physically adsorbed and residual water within the gel network [43]. During the second stage, a significant loss of mass (54.16% weight loss) occurred between 200 °C and 500 °C, which corresponded to the core stage of the chemical blowing process. Within this temperature range, the blowing agent (NH_4_Cl) underwent thermal decomposition to produce NH_3_ and HCl gases [44]. Simultaneously, glucose underwent dehydration and carbonization reactions. As the sintering temperature increased from 500 °C to 900 °C, the DSC curve showed a mass loss of approximately 26.69%, accompanied by a distinct exothermic trend. The carbon template (amorphous Al-Si@C) underwent oxidation during this stage, which generated CO_2_ [45]. Finally, when the temperature exceeded 900 °C, there was almost no mass loss, indicating that the oxidation-related removal of the carbonaceous component had been essentially completed. The differential scanning calorimetry (DSC) curve exhibited a sharp and distinct exothermic peak at around 1000 °C. This characteristic peak clearly indicated the phase transition of the material from an amorphous Al-Si network to crystalline mullite, as well as the formation of a highly crystalline mullite final product at temperatures above 1200 °C [46].

Figure 2b shows the phase transformation evolution of the precursor at key heat treatment temperatures (110 °C, 200 °C, 300 °C, 500 °C, and 1200 °C). At temperatures below 200 °C, the main phase was NH_4_Cl, indicating that the blowing agent remained stable at this stage. At 300 °C, the NH_4_Cl peak disappeared completely, and a broad peak emerged. This stemmed from the complete decomposition of NH_4_Cl, the consequent gas release, and the formation of an amorphous carbon network. This phenomenon corresponded to the substantial mass loss evident in the TG curve. When the temperature was further increased to 500 °C, the material maintained its broad amorphous peak, but the peak shifted to a smaller angle [47]. This shift can be attributed to the partial oxidation of the amorphous carbon network [48]. Finally, when the temperature reached 1200 °C, the amorphous peak disappeared, and the characteristic peak of highly crystalline mullite appeared [49].

The chemical bond evolution of the precursor was further elucidated by FTIR analysis (Figure 2c). When the temperature was below 200 °C, the intensity of the O–H stretching vibration peak weakened, indicating that the adsorbed water molecules had been released and a dehydration reaction occurred within the gel network [50]. Glucose began to carbonize at 300 °C, with the weakening of the C–H stretching peak and the strengthening of the C=O stretching peak [51]. Upon further increase to 500 °C, the intensity of the Si–O–Si vibrational peak intensified, while the C–H stretching band at approximately 2980 cm^−1^ and the band around 1590 cm^−1^ associated with conjugated C=C structures remained detectable, indicating the presence of the carbonaceous Al–Si@C intermediate. At 1200 °C, the characteristic organic peaks disappeared, and a sharp Al–O–Si vibration peak appeared, confirming that the carbon template had been completely oxidized and crystallized into pure mullite [46].

**Figure 3:**
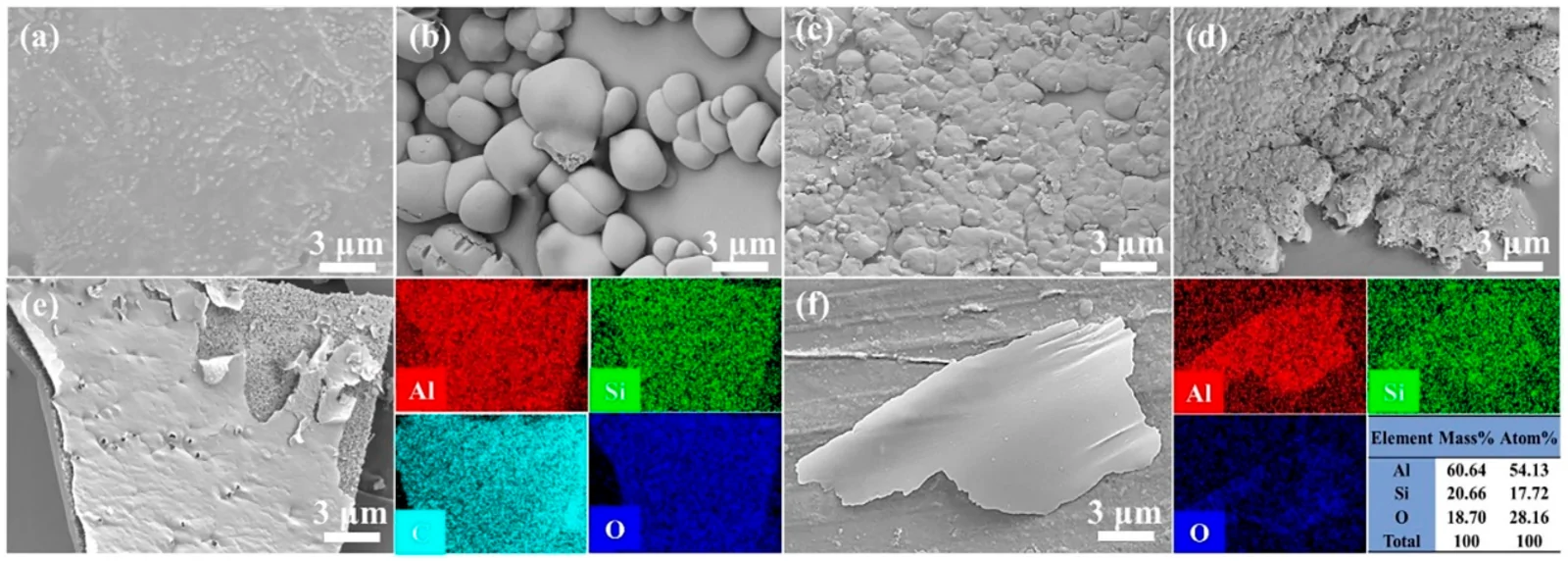
SEM images of precursors at different sintering temperatures of (**a**) 110 °C, (**b**) 200 °C, (**c**,**d**) 300 °C, (**e**) 500 °C and EDS elemental mapping scanning, (**f**) 1200 °C and corresponding elemental mappings.

To illustrate the morphological evolution associated with the aforementioned thermal and chemical reactions, SEM was performed on the precursors at different sintering temperatures (Figure 3). After 48 h of vacuum drying, the glucose/Al–Si precursor became a highly viscous solution, allowing the subsequently added NH_4_Cl particles to be effectively encapsulated within the matrix. At 110 °C (Figure 3a), further dehydration of the precursor occurred, while NH_4_Cl remained undecomposed and embedded within the glucose/Al–Si matrix, resulting in a compact morphology with a rough surface. As shown in Figure 3b, localized bubble aggregation was observed on the surface of the precursor at 200 °C. This result is consistent with the TG-DSC and XRD results, indicating that NH_4_Cl began to decompose, producing NH_3_ and HCl gases. However, the amount of gas released was limited, and most of the blowing agent remained stable. Thus, bubble formation had already been initiated at approximately 200 °C, when the glucose-containing matrix had not yet undergone pronounced carbonization. As the temperature increased from 200 to 300 °C, NH_4_Cl progressively decomposed and released gases, while the glucose/Al–Si precursor simultaneously underwent dehydration, condensation, and gradual carbonization. As the temperature increased to 300 °C (Figure 3c), a dense array of bubbles appeared on the surface of the precursor. At this temperature, the disappearance of the NH_4_Cl diffraction peaks indicated that its decomposition was essentially complete, while the FTIR results showed the onset of pronounced glucose carbonization. Therefore, the dense bubble arrays observed at 300 °C were formed progressively during the preceding gas-release process rather than being generated after complete solidification of the precursor. The in-situ generated gases accumulated and expanded within the precursor during the 200–300 °C interval, promoting the growth and interaction of adjacent bubbles and resulting in the dense bubble arrays observed at 300 °C. As shown in Figure 3d, the bubble array began to aggregate and merge, forming a layer-like structure with a large lateral dimension and an irregular shape. As the bubbles expanded and approached each other during the blowing process, the intervening precursor films were progressively compressed, thinned, and stretched, promoting their evolution toward ultrathin layered structures. As shown in Figure 3e, when the precursor was further heated to 500 °C, the array of bubbles previously observed evolved into a wide and ultrathin two-dimensional sheet-like structure. This morphological evolution occurred due to the continuous growth and expansion of the bubbles, leading to intense compression between adjacent bubble walls. This sustained compression exerted a significant tensile force on the liquid film between the bubbles, causing the walls to burst and stretch into an ultrathin layered structure [52,53]. At the same time, the further carbonization of glucose acted as a crucial rigid carbon template (i.e., Al–Si@C intermediate) that effectively stabilized the ultrathin two-dimensional structure. Furthermore, EDS element mapping at 500 °C revealed that the elements of Al, Si, O, and C were distributed over the entire sheet surface, indicating that the precursor had transformed into the Al-Si@C intermediate. After heat treatment at 1200 °C (Figure 3f), the amorphous carbon template was completely oxidized and removed in the form of carbon dioxide. The remaining amorphous aluminum-silicon network then underwent an in-situ phase transformation and fully crystallized. Therefore, the resulting mullite product exhibited a two-dimensional, sheet-like structure. The EDS spectrum at this temperature showed a uniform distribution of Al, Si and O, while the carbon signal had completely disappeared. The Al:Si atomic ratio was determined to be about 3:1, which was consistent with the theoretical stoichiometric ratio of mullite (3Al_2_O_3_·2SiO_2_). This indicated that the carbon template had been completely oxidized and removed, and the amorphous film after stretching successfully crystallized into mullite nanosheets.

**Figure 4:**
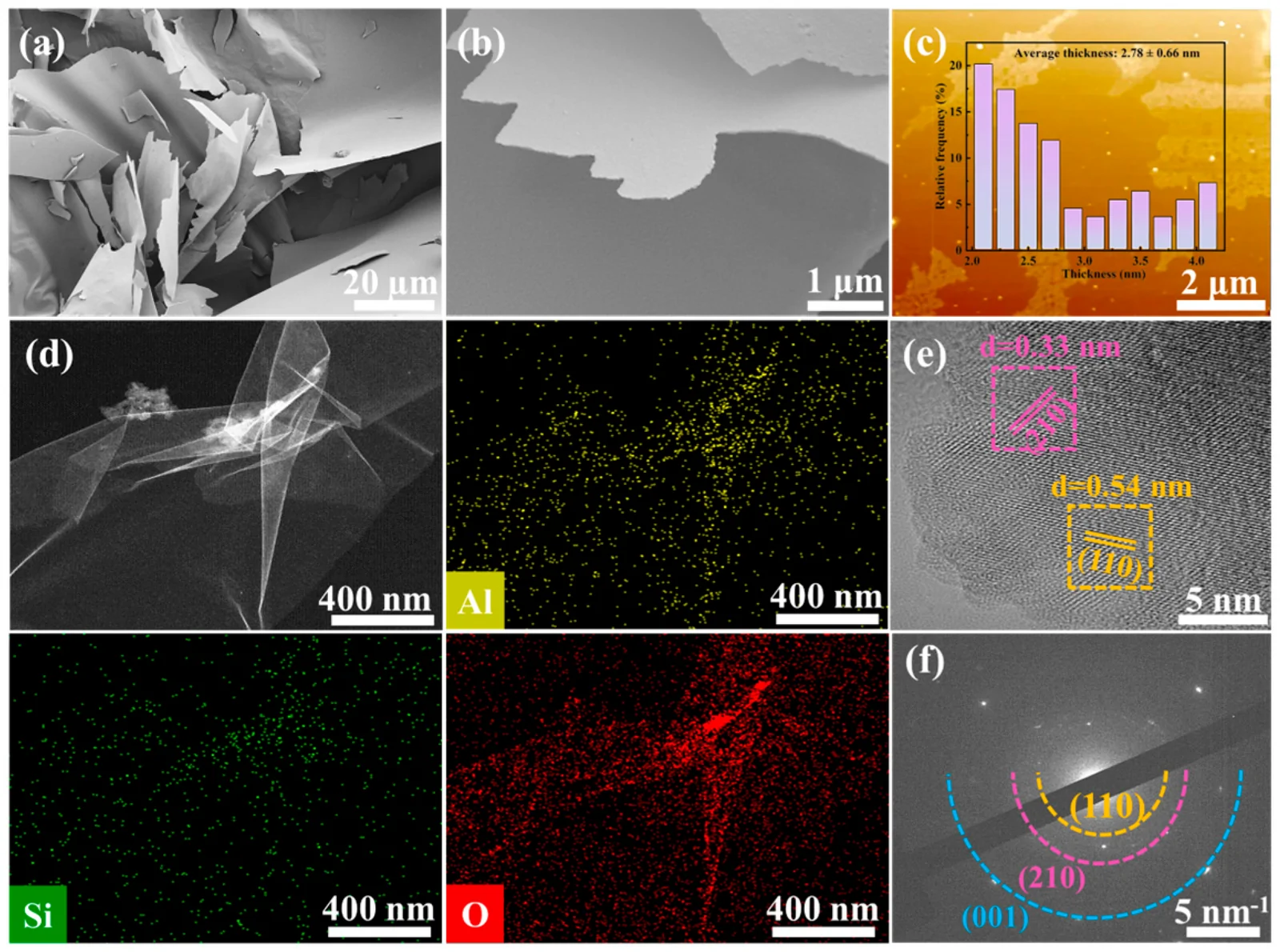
(**a**) SEM images of mullite nanosheets prepared at 1200 °C, (**b**) higher magnification of (**a**), (**c**) AFM images of mullite nanosheets and thickness distribution, (**d**) HAADF-STEM face-scan images of mullite nanosheets showing the uniform distribution of Si, O, and Al elements, (**e**) HR-TEM images of mullite nanosheets, (**f**) the corresponding SAED pattern.

The SEM and TEM images in Figure 4 showed the microstructure of the mullite nanosheets. As shown in the SEM images (Figure 4a,b), the material exhibited a curled nanosheet morphology with lateral dimensions of up to several hundred micrometers, indicating the high uniformity of the mullite nanosheets. Additionally, to provide a more statistically representative evaluation of the nanosheet thickness, a thickness distribution was added to Figure 4c. As shown in Figure 4c, the nanosheet thickness was mainly distributed within approximately 2–3 nm, with an average value of 2.78 ± 0.66 nm, further confirming the ultrathin characteristics of the prepared mullite nanosheets. The TEM image (Figure 4d) showed a translucent two-dimensional ultrathin structure composed of interconnected nanocrystals. The corresponding element mapping image revealed a uniform distribution of Al, Si, and O. As shown in Figure 4e, the HRTEM exhibited lattice fringes of 0.54 nm and 0.33 nm, consistent with the (110) and (210) crystal planes of mullite. The SAED pattern shown in Figure 4f showed distinct diffraction rings, indicating that the prepared mullite nanosheets had a polycrystalline structure. The detailed observations using SEM and TEM (Supporting Information, Figure S4) further revealed that the nanosheets exhibited a continuous sheet-like morphology with a relatively smooth surface.

**Figure 5:**
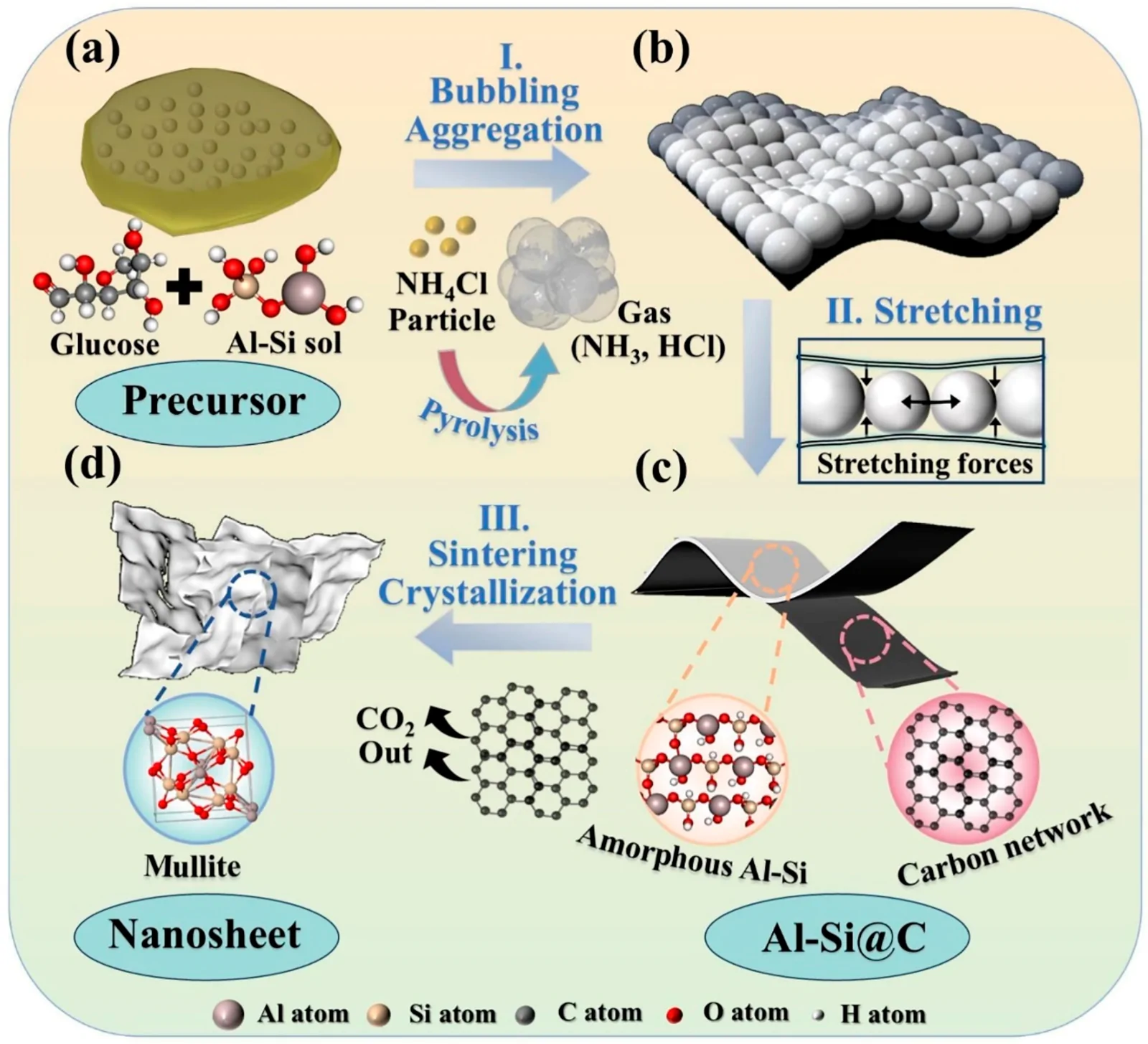
Schematic diagram of the blowing mechanism for preparing ultrathin mullite nanosheets: (**a**) precursor formation, (**b**) bubble aggregation and stretching, (**c**) formation of the Al–Si@C intermediate and (**d**) formation of mullite nanosheets.

As shown in Figure 5, the formation mechanism of the ultrathin mullite nanosheets was driven by a chemical blowing strategy. This schematic specifically highlights the critical synergistic role played by glucose and NH_4_Cl. As shown in Figure 5a, the precursor solution consisted of Al-Si sol and glucose. As the solvent water gradually evaporated under vacuum drying, the solute concentration and solution viscosity increased accordingly. Therefore, the subsequently added NH_4_Cl was encapsulated within the viscous solution. As the temperature increased, the blowing agent (NH_4_Cl) in the precursor matrix underwent thermal decomposition, producing large amounts of NH_3_ and HCl gases. As shown in Figure 5b, these in-situ generated gases expanded within the matrix, forming a large number of bubbles. These bubbles were evenly and closely arranged within the matrix, forming a regular two-dimensional bubble array. As the amount of gas increased (Figure 5c), the bubbles continued to expand and move closer together. The bubble walls were subjected to pressure due to compression between the bubbles. This forces the liquid films between adjacent bubbles to undergo planar stretching, ultimately transforming into ultrathin sheet-like structures. This ultrathin structure was unstable due to its extremely high surface energy. At this temperature, glucose underwent an in-situ carbonization reaction and transformed into a rigid three-dimensional carbon network framework. This rigid carbon network framework provided physical support for the unstable aluminum-silicon film, enabling it to form a stable layered Al–Si@C intermediate structure (Figure 5c). With increasing temperature during air sintering, the originally rigid carbon template was completely oxidized and removed, leaving only an amorphous aluminum-silicon network. This network underwent further phase transformation and in-situ crystallization reactions at high temperatures, eventually transforming into ultrathin and highly crystalline mullite nanosheets (Figure 5d).

### 3.2. Preparation of Hierarchically Mullite Porous Ceramics

#### 3.2.1. Effect of Solid Content on Porous Ceramics

A three-dimensional hierarchical mullite porous ceramic was prepared by combining gel casting and freeze-drying processes. As shown in Figure 1b, this process used ground mullite nanosheets as the basic building unit, agarose as the gelling agent, and silica sol as the high-temperature binder. After grinding and dispersion, partial fragmentation and local cracking of the nanosheets were observed (Supporting Information, Figure S1). Nevertheless, the powders retained their two-dimensional sheet-like morphology, although their lateral dimensions were reduced. These smaller flakes could still overlap and interlock during gel casting to construct the three-dimensional porous framework.

**Figure 6:**
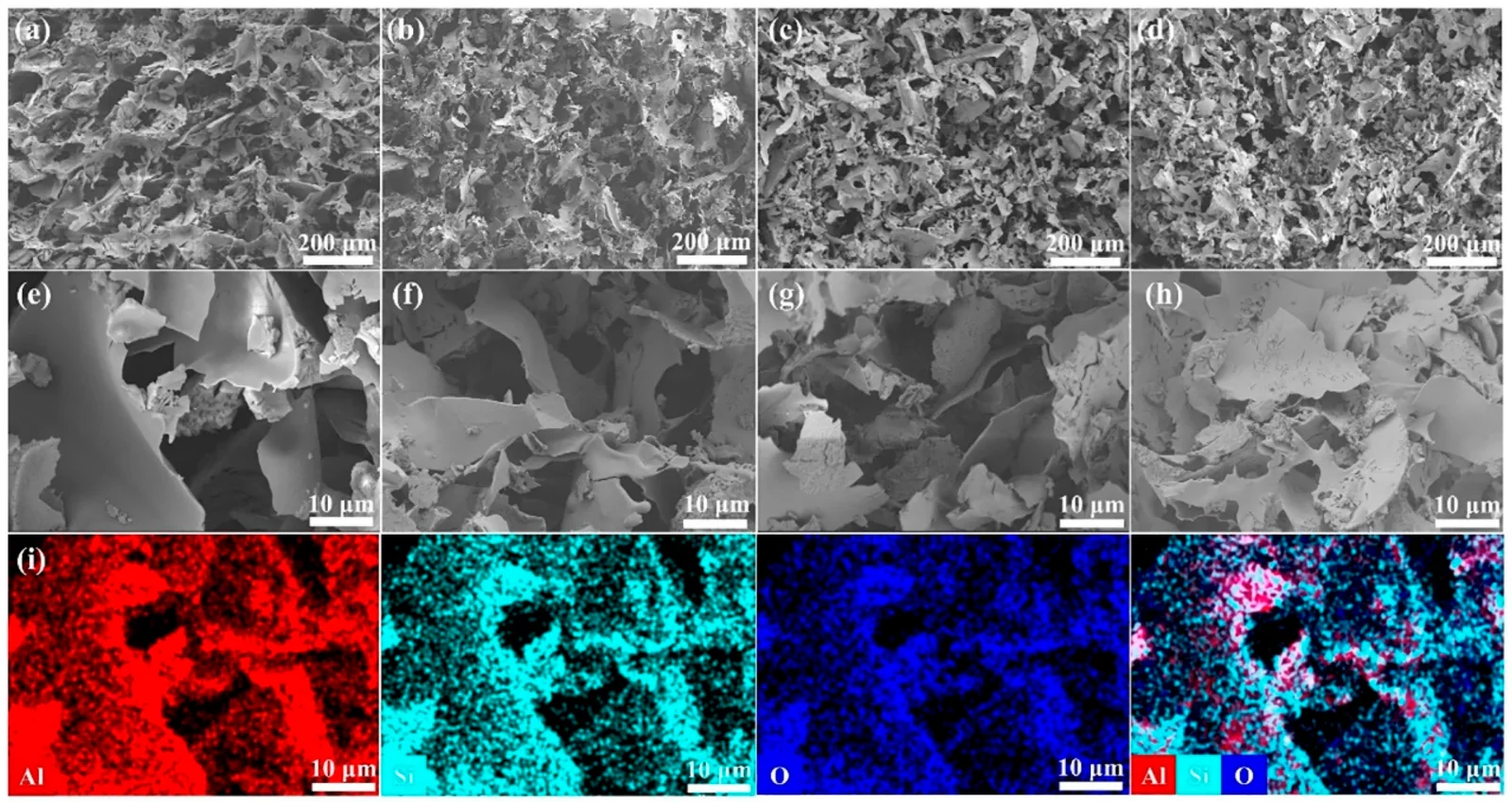
SEM images of mullite porous ceramic sintered at 1200 °C with different solid contents (**a**) 10 wt.%, (**b**) 15 wt.%, (**c**) 20 wt.%, (**d**) 25 wt.%, (**e**–**h**) high magnification of (**a**–**d**), respectively, (**i**) corresponding Al, Si and O elemental distribution maps of (**e**).

Figure 6 shows the effect of solids content on the microstructure of mullite porous ceramics. At a solids content of 10 wt.% (Figure 6a), the sample exhibited large pores with a coarse and irregular morphology. Figure 6e showed that the mullite nanosheets were sparsely distributed and could not interlock effectively during freeze-drying. This sparse distribution failed to inhibit ice crystal growth, resulting in larger pores and thereby weakening the mechanical strength of the overall framework. The EDS spectrum (Figure 6i) revealed a homogeneous distribution of Al, Si, and O elements on the nanosheets. When the solid content reached 15 wt.% (Figure 6b), the pore size significantly decreased and a regular structure was observed [54]. As shown in Figure 6f, the mullite nanosheets began to overlap and interlock with each other, forming a more continuous network structure. This interlocking structure effectively inhibited excessive ice crystal growth and reduced pore size [55]. When the solid content further increased to 20 wt.%, (Figure 6c), the macropores resulting from freeze-drying became significantly smaller and more uniform. As shown in Figure 6g, the mullite nanosheets exhibited a dense interlocking structure. The dense interlocking and overlapping of the nanosheets had created numerous secondary micropores, giving the material a hierarchical porous structure [56]. However, at a solid content of 25 wt.% (Figure 6d), the increased nanosheet content excessively suppressed ice crystal growth, resulting in a locally densified and unevenly distributed pore structure. As shown in Figure 6h, a high content of mullite nanosheets resulted in stacking and agglomeration, causing some pores to close.

**Figure 7:**
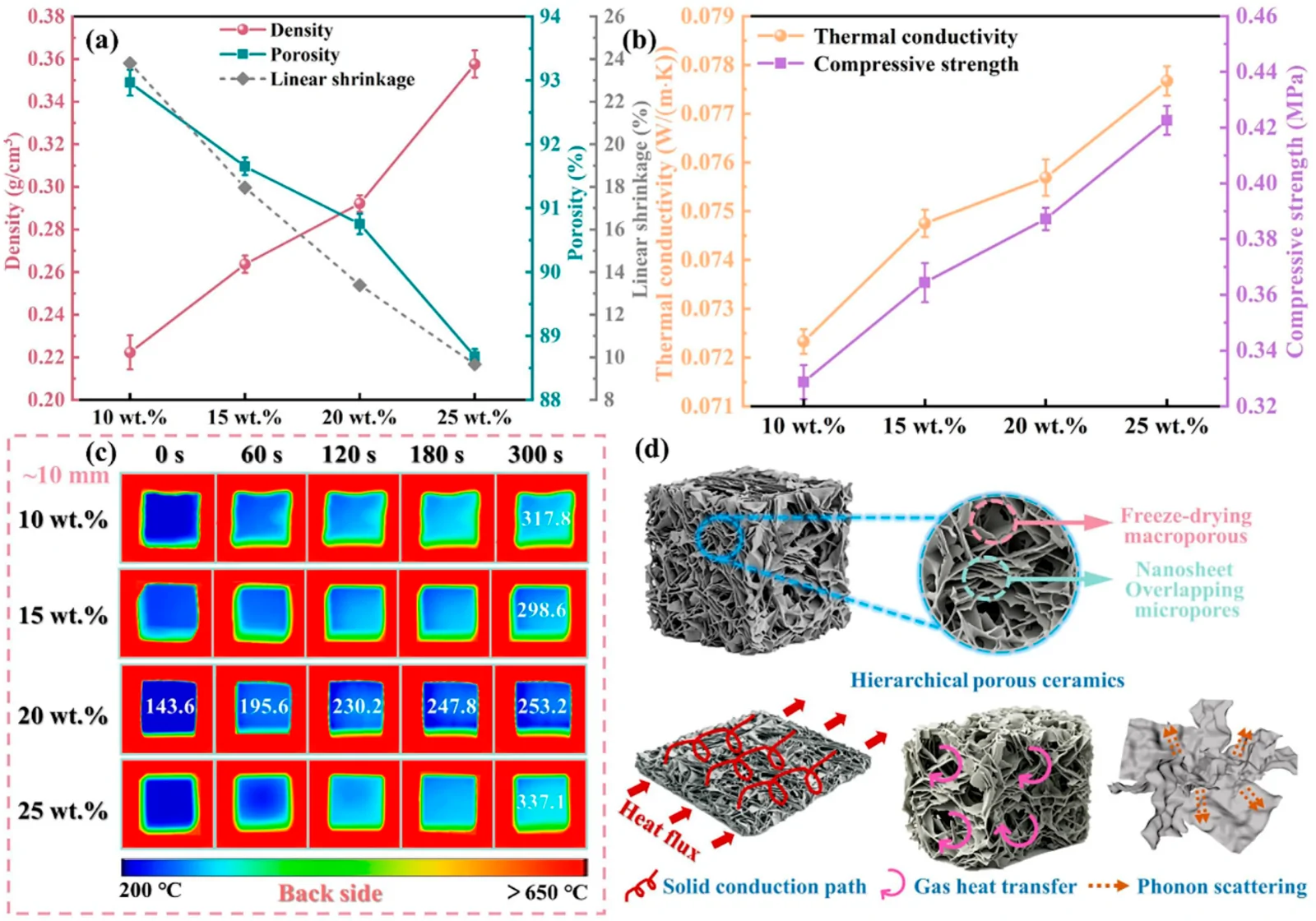
(**a**) Bulk density and apparent porosity of the porous ceramics at different solid contents, (**b**) the effect of solid content on the apparent thermal conductivity and compressive strength of the porous ceramics, (**c**) the infrared images of mullite porous ceramics with different solid contents, (**d**) schematic illustration of the thermal insulation performance of porous ceramics.

Figure 7a shows the bulk density, apparent porosity, and linear shrinkage of porous ceramics at different solid contents. As the solid content increased from 10 wt.% to 25 wt.%, the density of the porous ceramic increased from 0.223 g/cm^3^ to 0.358 g/cm^3^. Conversely, the porosity decreased from 92.10% to 88.28%. This was attributed to the gradual increase in the content of solid mullite nanosheets, which promoted tighter bonding and stacking within the material, thereby increasing the density and reducing the porosity. As the solid content increased, the linear shrinkage rate gradually decreased from 23.81% to 9.68%. This was due to the formation of a more rigid three-dimensional network structure at higher solid content. This structure can effectively resist the sintering driving force, resulting in reduced linear shrinkage.

The effect of solid content on the apparent thermal conductivity and compressive strength of mullite porous ceramics is shown in Figure 7b. When the solid content increased from 10 wt.% to 25 wt.%, the thermal conductivity of the sample at room temperature increased from 0.0723 W/(m·K) to 0.0776 W/(m·K). Theoretically, heat transfer in porous media involves conduction through the solid skeleton and pore gas, convection associated with fluid motion within the pores, and thermal radiation, with their relative contributions depending on the pore structure, fluid properties, temperature, and boundary conditions [57,58]. For the highly porous ceramics, the small pore dimensions and the absence of forced gas flow are expected to limit convective heat transfer, while the radiative contribution is relatively small at room temperature. Therefore, the apparent thermal conductivity at room temperature is mainly determined by the conductance of solids and gases, and it depends not only on porosity and volume density but also on the morphology and connectivity of the solid framework. When the solid content increased from 10 wt.% to 25 wt.%, the thermal conductivity gradually increased, which is consistent with the changes in density and porosity resulting from the increase in solid content.

The compressive strength increased from 0.33 to 0.42 MPa as the solid content was increased from 10 wt.% to 25 wt.%, consistent with the evolution of the microstructure and the trend in density changes. The increase in solid content led to higher density, thereby providing a more rigid three-dimensional framework that could better withstand external stress. When the solid content was 10 wt.%, the material formed a fragile three-dimensional network composed of loosely connected nanosheets. At higher solid contents (20–25 wt.%), it transformed into a highly dense and rigid framework structure. Therefore, this led to an increase in the compressive strength.

The high-temperature shielding performance of the porous ceramics was evaluated experimentally by exposing a 10 mm thick sample to a butane flame for 300 s, as shown in Figure 7c. All samples experienced a rapid temperature rise within 60 s, after which the backside temperature gradually approached a stable value at approximately 120 s. As the solid content increased from 10 wt.% to 25 wt.%, the stabilized backside temperature first decreased and then increased. Specifically, it decreased from 317.8 °C at 10 wt.% to 253.2 °C at 20 wt.%, and then gradually increased to 337.1 °C at 25 wt.%.

The thermal insulation mechanism of mullite porous ceramics at high-temperature conditions is schematically shown in Figure 7d. As discussed earlier, convective heat transfer is generally negligible in porous ceramics [59,60]. At high temperatures, solid and gas conduction remain important, while the contribution of thermal radiation increases significantly with increasing temperature and becomes increasingly important [43]. Therefore, its heat insulation performance mainly depended on the combined regulation of solid conduction, gas conduction, and thermal radiation by the hierarchical three-dimensional structure. At 10 wt.% solid content, the material exhibited large pores that provided direct pathways for the heat flux, enhancing gas heat transfer. Because the low solid content and loosely interlocking nanosheet network failed to suppress infrared radiation, leading to a rapid increase in the backside temperature. Increasing the solid content to 15 wt.%, the pore size decreased significantly and a regular pore structure emerged. This not only extended the gas diffusion path but also the solid conduction path, thereby inhibiting gas and solid heat transfer. However, the low solid content limited the ability of the nanosheet network to scatter intense thermal radiation, resulting in the radiation heat transfer remaining at a relatively high level. At a solid content of 20 wt.%, the excellent high-temperature insulation performance of porous ceramics was mainly attributed to the formation of an interconnected three-dimensional nanosheet network and hierarchical pore structure. As shown in Figure S3 (Supporting Information), distinct characteristic peaks were observed at diameters of approximately 2.9 μm and 6.6 μm. Additionally, there were also relatively weak diameter distributions within the range of approximately 100 μm to 300 μm, indicating the presence of hierarchical pores within the sample. Meanwhile, the specific surface area of the sample reached 5.007 m^2^/g, indicating that it has a large surface area and abundant solid–gas interfaces. In high-temperature environments, the abundant solid–gas interfaces transformed into highly efficient scattering and absorption centers, resulting in multiple reflections and dissipation of thermal radiation, thereby significantly reducing the intensity of thermal radiation. At the same time, the secondary micropores greatly restricted the free movement of gas molecules, significantly weakening the gas heat transfer. This structure also effectively prevented the formation of continuous solid heat conduction pathways, further reducing solid heat transfer, and thus achieving the lowest backside temperature. However, as the solid content further increased to 25 wt.%, the high-temperature thermal insulation performance decreased. This was because the stacking and agglomeration of nanosheets disrupted the original regular microporous structure. This agglomeration phenomenon formed a continuous solid thermal bridge within the material. Heat flux was conducted directly through this solid thermal bridge, shortening and simplifying the heat transfer pathways and leading to a significant increase in the back-side temperature [61].

The above results demonstrate that the 20 wt.% sample achieved a favorable combination of high porosity, thermal insulation performance, and mechanical integrity. These results indicate that the interconnected nanosheet framework maintains structural integrity while retaining the high porosity and low thermal conductivity required for thermal insulation. Therefore, 20 wt.% was selected as the optimal solid content for subsequent investigation.

#### 3.2.2. The High-Temperature Stability of Porous Ceramics

The structural integrity and thermal stability of the material are critical for high-temperature applications. Therefore, this work further investigated the phase stability, microstructural evolution, and comprehensive performance of the porous ceramics with a solid content of 20 wt.% at temperatures ranging from 1200 °C to 1500 °C.

**Figure 8:**
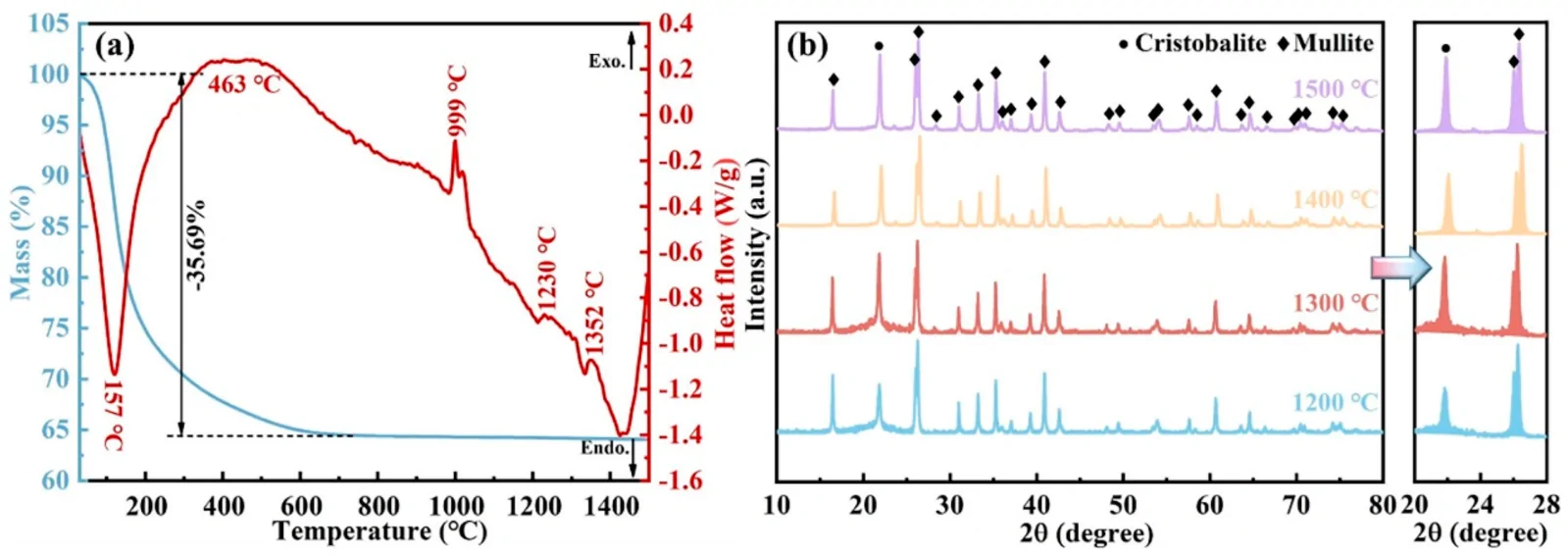
(**a**) TG-DSC curves of the porous ceramics at 25–1500 °C under an air atmosphere, (**b**) XRD patterns of the samples sintered at 1200–1500 °C and the sample 20° to 28°.

To clarify the high-temperature stability of porous ceramics, the thermal behavior of the green bodies was analyzed by TG-DSC from room temperature to 1500 °C under an air atmosphere. As shown in Figure 8a, the total mass loss of the sample during the entire heating process was approximately 35.69%, which was completed before 800 °C. The evaporation of physically adsorbed water and residual solvents caused the rapid mass loss and endothermic peak observed from room temperature to 200 °C. Between 200 °C and 600 °C, a further mass loss corresponded to a broad exothermic peak centered at 463.0 °C, originating from the oxidation and thermal decomposition of the organic components, including the agarose used as the gelling agent. The mass became essentially stable before 800 °C, indicating that the organic constituents had been removed before the high-temperature sintering stage. At 999 °C, a distinct exothermic peak was observed without any accompanying mass loss, which was mainly attributed to the crystallization of silica sol derived into cristobalite. The slight endothermic peaks at 1230 °C and 1352 °C were related to the softening of localized glass phases and the sintering densification of the ceramic. Therefore, above 1200 °C, no further loss of mass occurred, confirming the high-temperature stability of the porous ceramic.

The XRD patterns clearly showed the phase transformation of the porous ceramics at different sintering temperatures, as shown in Figure 8b. Within the temperature range of 1200 °C to 1500 °C, the phase composition of the sample was all mullite (PDF#79-1545) and cristobalite (PDF#85-0512). It should be noted that the cristobalite originated from the small amount of silica sol used as an inorganic binder, whereas the main structural framework remained predominantly composed of interconnected mullite nanosheets. As the temperature increased from 1200 °C to 1500 °C, no other phases or amorphous peaks were detected, but the intensity of the characteristic diffraction peaks increased significantly. In the 20° to 28° diffraction region, the characteristic diffraction peaks of cristobalite (2θ ≈ 22°) and mullite (2θ ≈ 26°) gradually became sharper and narrower. This enhancement of the diffraction peak was attributed to the higher sintering temperature, which promoted grain growth and structural ordering. The results indicated that porous ceramics exhibit phase stability under high-temperature conditions.

**Figure 9:**
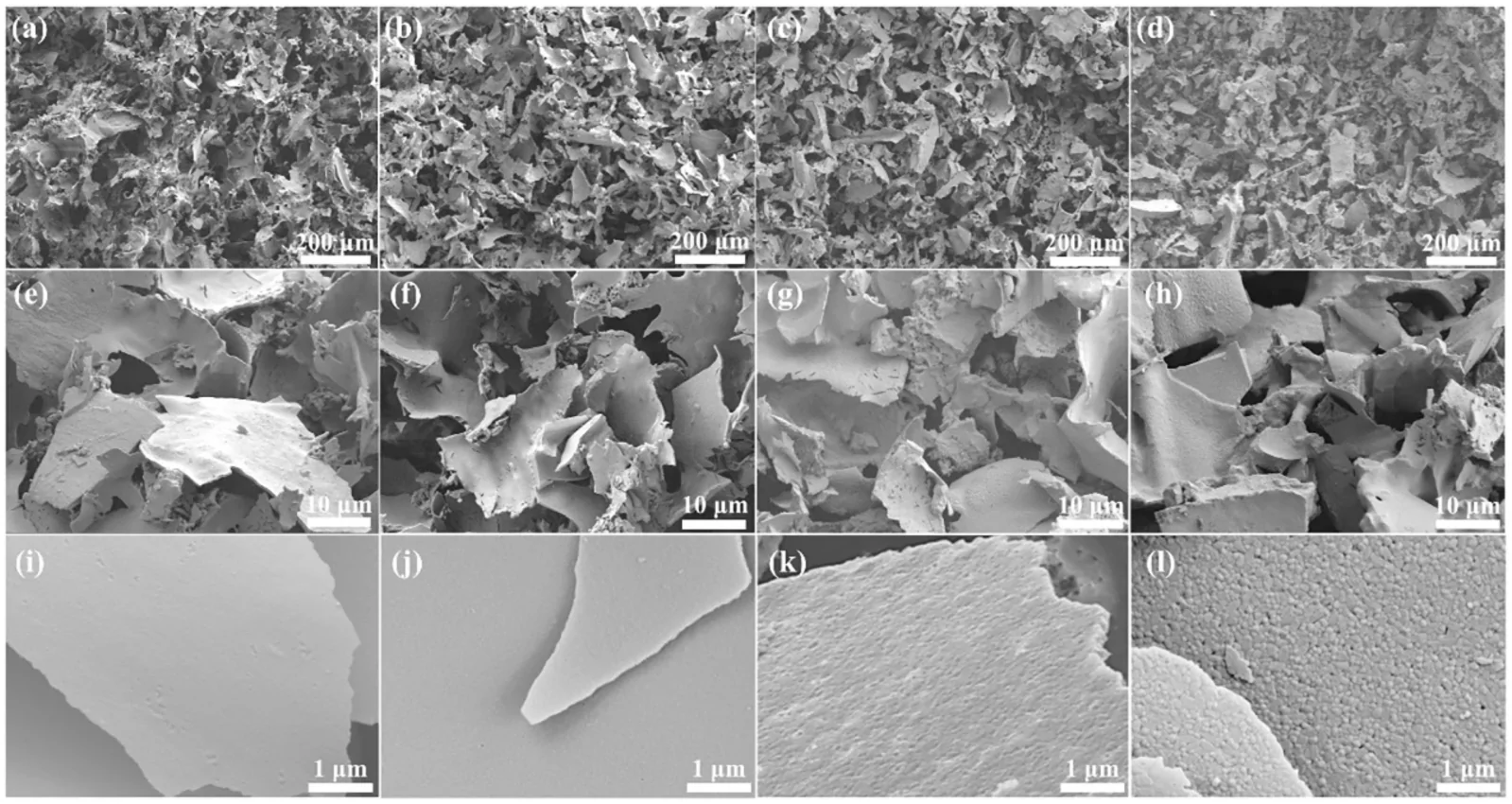
(**a**–**d**) SEM images of the porous ceramics sintered at (**a**) 1200 °C, (**b**) 1300 °C, (**c**) 1400 °C, (**d**) 1500 °C, (**e**–**h**) high-magnification of (**a**,**b**), respectively, (**i**–**l**) high-magnification of (**e**–**h**), respectively.

Figure 9 shows the evolution in the microstructure of porous ceramics sintered at high temperatures ranging from 1200 °C to 1500 °C. At 1200 and 1300 °C, the ceramics exhibited a clear and regular hierarchical porous network structure with no significant shrinkage (Figure 9a,b). Meanwhile, the mullite nanosheets remained in a two-dimensional ultrathin morphology and an interlocked structure (Figure 9e,f). As shown in Figure 9i,j, the nanosheet surfaces were smooth and there were no obvious grain boundaries, indicating that no densification and grain growth had occurred. As the temperature increased to 1400 °C (Figure 9c), the interconnected, large-pore structure was preserved, but a degree of contraction was observed. However, the interconnected nanosheets exhibited grain growth and thickening, and significant necking was observed at the contact points, leading to the closure of the secondary micropores (Figure 9g). As shown in Figure 9k, the originally smooth sheet-like surface was completely replaced by countless small particles with clear boundaries. This microstructural evolution was attributed to the increased thermal energy, which accelerated local atomic diffusion and promoted the growth of mullite phase grains. Meanwhile, the cristobalite phase acted as a high-temperature binder, interconnecting adjacent mullite nanosheets. When the sintering temperature was further increased to 1500 °C (Figure 9d), the interconnected large pores did not collapse or undergo severe deformation, but local densification and further dimensional shrinkage occurred. As shown in Figure 9h, the interlocking two-dimensional mullite nanosheets became thicker and fused. As shown in Figure 9l, due to intensified sintering, the small grains on the surface of the nanosheets grew and merged into larger grains, which exhibited irregular arrangements and very distinct boundaries. At 1500 °C, the mullite did not melt, while the softened cristobalite encapsulated the coarsened mullite nanosheets, maintaining their three-dimensional porous structure. Despite localized densification and particle coarsening, this structure effectively prevented structural collapse. Therefore, the material successfully retained its hierarchical pore structure at 1500 °C without collapsing, demonstrating good structural stability under the high-temperature treatment conditions investigated in this study.

To elucidate the influence of sintering temperature on the performance of porous ceramics, samples sintered at 1200–1500 °C were systematically tested and characterized.

**Figure 10:**
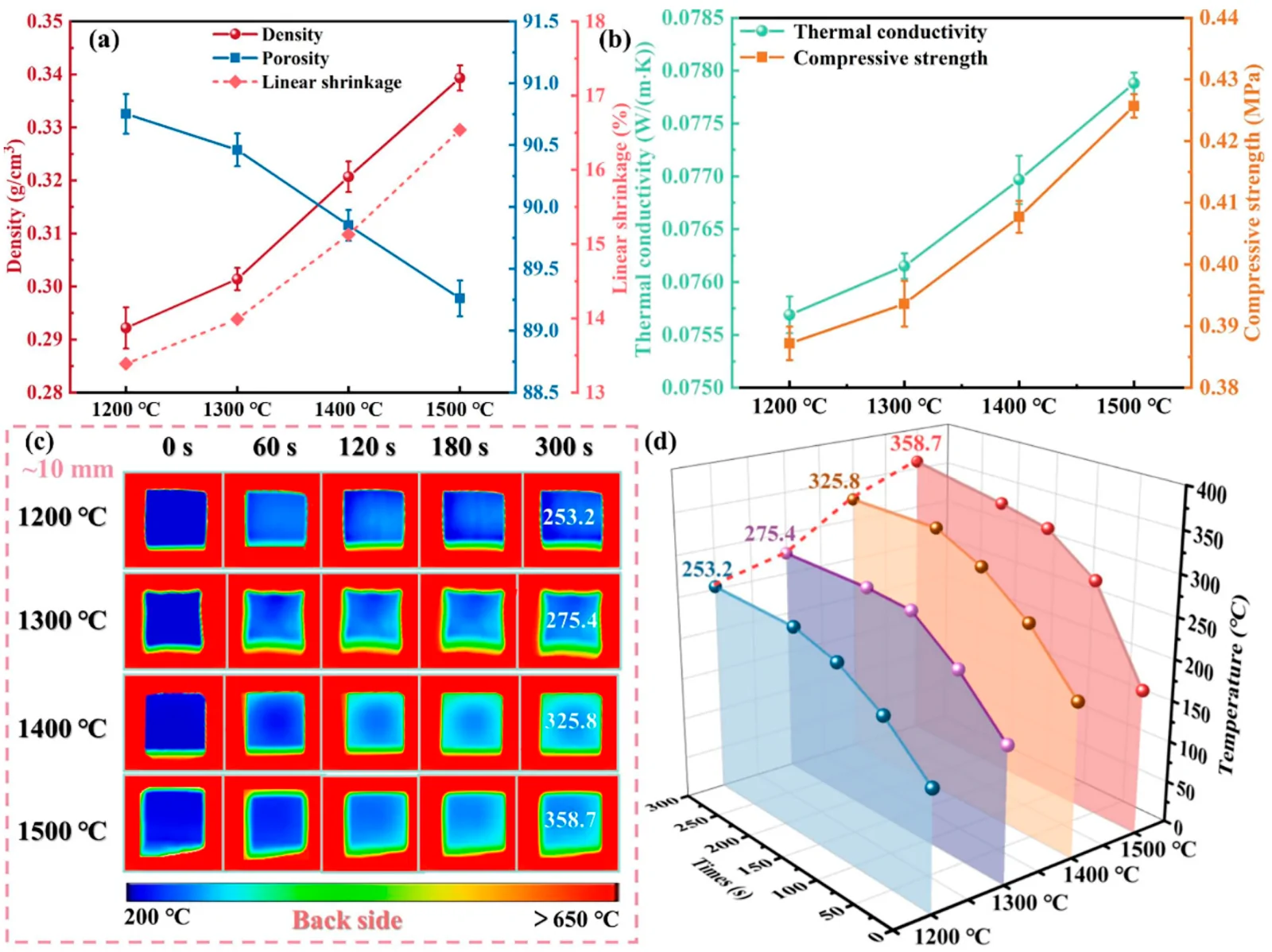
(**a**) Bulk density and apparent porosity of the porous ceramics at different sintering temperatures, (**b**) apparent thermal conductivity and compressive strength of the porous ceramics at different sintering temperatures, (**c**) infrared thermograms of the mullite porous ceramics sintered at various temperatures, (**d**) the corresponding backside temperature evolution curves for the specimens depicted in panel (**c**).

Figure 10a shows the bulk density, apparent porosity, and linear shrinkage of porous ceramics at different sintering temperatures. As the sintering temperature increased from 1200 °C to 1500 °C, the density and linear shrinkage rate of the porous ceramic increased gradually from 0.292 g/cm^3^ and 13.3% to 0.339 g/cm^3^ and 16.5%, respectively. Conversely, the porosity decreased from 90.75% to 89.26%. As the sintering temperature increased, the enhanced densification of the material led to simultaneous increases in both density and linear shrinkage. At the same time, porosity showed a corresponding decline, as confirmed by the SEM observations in Figure 9.

Figure 10b shows the evolution of compressive strength and apparent thermal conductivity of mullite porous ceramics with sintering temperature. From 1200 °C to 1500 °C, the compressive strength and the thermal conductivity exhibited a continuous rise trend with increasing sintering temperature. Specifically, compressive strength significantly increased from 0.39 MPa to 0.43 MPa, and thermal conductivity increased from 0.0756 W/(m·K) to 0.0779 W/(m·K). As mentioned above, the room-temperature thermal conductivity exhibited a dependence on material density, following a consistent trend with the density variation. The improvement in compressive strength was attributed to an increase in the sintering degree of the materials. Specifically, the sintering process promoted the growth and thickening of the mullite nanosheets and the formation of neck-like connections between adjacent particles. Consequently, this highly interconnected and rigid skeleton was capable of withstanding higher external loads.

As shown in Figure 10c,d, the thermal insulation performance of the sample was further evaluated by exposing it directly to a butane flame for 300 s. As the sintering temperature increased from 1200 °C to 1500 °C, the stabilized backside temperature gradually increased from 253.2 °C to 358.7 °C, and the corresponding thermal equilibrium time increased from 120 s to 180 s. As mentioned above, heat transfer in porous ceramics at high temperatures mainly occurs through thermal radiation. The samples sintered at 1200 °C exhibited a regular hierarchical pore structure and had the highest porosity. This structure provided abundant solid–gas interfaces, which could serve as highly effective scattering centers for the efficient attenuation and absorption of intense infrared radiation. Meanwhile, the small pores formed by the interlocking of thinner nanosheets extended the paths for heat conduction between the solid and gas, resulting in the sample having the lowest back temperature and excellent high-temperature insulation properties. As the sintering temperature increased to 1500 °C, grain coarsening and solid-state neck growth were significantly promoted. The merging and thickening of the nanosheets led to the formation of closed pores in part of the original hierarchical pore structure, which weakened thermal radiation scattering and enhanced solid-state heat conduction, thereby increasing the thermal equilibrium temperature.

To further evaluate the comprehensive performance of the nanosheet-assembled porous ceramic, its key properties were compared with representative porous mullite ceramics reported in the literature, as summarized in Table 1.

As shown in Table 1, existing porous mullite ceramics exhibit distinct advantages in individual properties, but trade-offs remain among porosity, mechanical strength, and thermal insulation. Nanofibrous ceramics and aerogels generally possess high porosity and low thermal conductivity but relatively limited mechanical strength [5,12,13], whereas freeze-cast, nanoparticle-stabilized, and gel-cast ceramics exhibit substantially higher compressive strengths at the expense of lower porosity and/or higher thermal conductivity [62,63,64]. Whisker- and fiber-based ceramics also demonstrate excellent high-temperature thermal insulation, but their overall property combinations remain constrained to different extents [50,65,66]. In comparison, the nanosheet-assembled ceramic developed in this work simultaneously achieves a porosity of 90.75%, a thermal conductivity of 0.0756 W/(m·K), and a compressive strength of 0.39 MPa, while maintaining a backside temperature of only 253.2 °C after exposure to a >1200 °C flame for 300 s and structural stability up to 1500 °C. These results demonstrate that the nanosheet assembly strategy provides a promising route for balancing lightweight characteristics, thermal insulation, and high-temperature stability in porous mullite ceramics.

## 4. Conclusions

In this work, hierarchically porous mullite ceramics assembled from two-dimensional ultrathin nanosheets were successfully synthesized to achieve a favorable balance between thermal insulation and mechanical integrity at high porosity. The formation mechanism, structural evolution, and high-temperature behavior were investigated, and the main conclusions are as follows:

(1) Highly crystalline mullite nanosheets with a thickness of only about 2–3 nm was successfully prepared through a chemical blowing strategy based on the synergistic interaction between glucose and NH_4_Cl.
(2) Through the gel casting and freeze-drying processes, the nanosheets were assembled into a rigid hierarchical pore network connected by a silica sol binder. An optimal solid content of 20 wt.% achieved an excellent balance between suppressing ice crystal formation and ensuring nanosheet dispersal.
(3) This hierarchical structure not only acts as an efficient scattering center for infrared radiation but also extends the heat conduction paths in solids and gases. As a result, this ceramic material exhibited a high porosity of 90.75%, a compressive strength of 0.39 MPa, and excellent thermal insulation properties.

## Figures and Tables

**Table 1:** Comparison of the comprehensive properties of porous mullite ceramics.

Type	Ref.	ρ (g/cm^3^)	P (%)	σ (MPa)	λ_RT_ W/(m·K)	High-Temperature Insulation Performance	Thermal Stability
Nanofibrous ceramic	[5]	0.202	≈96	0.837	0.0715	—	—
Nanofibrous aerogel	[12]	0.0346–0.0489	—	—	0.0327–0.0432	—	1400
Hollow-sphere particles	[13]	—	79.9–88.7	0.20–6.70	—	—	—
Whiskers	[50]	0.76	74.18	4.81	0.260	T_b_ < 200 °C	1300
Freeze casting	[62]	0.49–1.23	60.2–83.4	3.8–49.4	0.18	—	—
Nanoparticle stabilized foam	[63]	—	64.0–87.0	6.6–40.4	≥0.10	—	—
Gel casting	[64]	—	75.85	14.02	0.263	λ_1000_ = 0.544	1000
Micro/nanofiber structure	[65]	0.50	—	1.43	—	T_b_ = 355.8 °C	900
Near-net-size structure	[66]	—	84.2	2.45 ± 0.15	0.19	ΔT = 800 °C	—
Nanosheets	This work	0.292	90.75	0.39	0.0756	T_b_ = 253.2 °C	1500

Note: —, not reported.

## Data Availability

The original contributions presented in this study are included in the article. Further inquiries can be directed to the corresponding authors.

## Supplementary Materials

The following supporting information can be downloaded at: https://www.mdpi.com/article/10.3390/ma19163433/s1, Figure S1: (a) SEM images of mullite nanosheets after milling: overall morphology, (b) High magnification of (a); Figure S2. (a) Schematic illustration of the butane-flame thermal-insulation test, (b) infrared thermogram of the flame-facing surface measured under the same flame-exposure conditions; Figure S3: Pore structure distribution of the 20 wt.% porous ceramic sintered at 1200 °C; Figure S4. Morphological characterization of the ultrathin mullite nanosheets: (a,b) SEM image, (c,d) TEM image; Figure S5. SEM images showing the structural evolution of mullite nanosheet-assembled porous ceramics during processing: (a) chemical blowing, (b) freeze-drying, (c) sintering.

## References

[1] Zhang Q., Huang H., Lei C., Liu Y., Li W. (2025). Review of Lightweight, High-Temperature Thermal Insulation Materials for Aerospace. Materials.

[2] Liu L., Wang X., Zhang Z., Shi Y., Zhao Y., Shen S., Yao X., Shen J. (2022). A Facile Method for Fabricating a Monolithic Mullite Fiber-Reinforced Alumina Aerogel with Excellent Mechanical and Thermal Properties. Gels.

[3] Zhao K., Ye F., Cheng L., Yang J., Chen X. (2023). An overview of ultra-high temperature ceramic for thermal insulation: Structure and composition design with thermal conductivity regulation. J. Eur. Ceram. Soc..

[4] Zhang Y., Wu Y., Yang X., Li D., Zhang X., Dong X., Yao X., Liu J., Guo A. (2020). High-strength thermal insulating mullite nanofibrous porous ceramics. J. Eur. Ceram. Soc..

[5] Almeida R., Bergmueller E., Luehrs H., Wendschuh M., Clauss B., Tushtev K., Rezwan K. (2017). Thermal exposure effects on the long-term behavior of a mullite fiber at high temperature. J. Am. Ceram. Soc..

[6] He J., Li X., Su D., Ji H., Wang X. (2016). Ultra-low thermal conductivity and high strength of aerogels/fibrous ceramic composites. J. Eur. Ceram. Soc..

[7] Schneider H., Schreuer J., Hildmann B. (2008). Structure and properties of mullite—A review. J. Eur. Ceram. Soc..

[8] Mahnicka-Goremikina L., Svinka R., Svinka V., Grase L., Juhnevica I., Rundans M., Goremikins V., Tolendiuly S., Fomenko S. (2022). Thermal Properties of Porous Mullite Ceramics Modified with Microsized ZrO_2_ and WO_3_. Materials.

[9] Han F., Zhong Z., Yang Y., Wei W., Zhang F., Xing W., Fan Y. (2016). High gas permeability of SiC porous ceramics reinforced by mullite fibers. J. Eur. Ceram. Soc..

[10] Wang Y., Liu H.T., Cheng H.F., Wang J. (2016). Application of Sol-Gel Derived Mullite in Three-Dimensional Oxide Fiber-Reinforced Composites as Porous Matrix. Rare Metal. Mat. Eng..

[11] Liu R., Dong X., Xie S., Jia T., Xue Y., Liu J., Jing W., Guo A. (2019). Ultralight, thermal insulating, and high-temperature-resistant mullite-based nanofibrous aerogels. Chem. Eng. J..

[12] Chen A., Li M., Xu J., Lou C., Wu J., Cheng L., Shi Y., Li C. (2018). High-porosity mullite ceramic foams prepared by selective laser sintering using fly ash hollow spheres as raw materials. J. Eur. Ceram. Soc..

[13] Choo T., Mohd Salleh M., Kok K., Matori K. (2019). Modified cenospheres as non-sacrificial pore-forming agent for porous mullite ceramics. Ceram. Int..

[14] Shao Y., Lin L., Xu J., Wang H., Xia C., Feng X., Gao F. (2024). Near-zero sintering shrinkage porous mullite ceramics with high porosity and low thermal conductivity. Ceram. Int..

[15] Yang Z., Yang F., Zhao S., Li K., Chen J., Fei Z., Chen G. (2021). In-situ growth of mullite whiskers and their effect on the microstructure and properties of porous mullite ceramics with an open/closed pore structure. J. Eur. Ceram. Soc..

[16] Smith D.S., Martinez Dosal J., Oummadi S., Nouguier D., Vitiello D., Alzina A., Nait-Ali B. (2022). Neck formation and role of particle-particle contact area in the thermal conductivity of green and partially sintered alumina ceramics. J. Eur. Ceram. Soc..

[17] Hu L., Wang C., Hu Z., Lu S., Sun C., Huang Y. (2011). Porous yttria-stabilized zirconia ceramics with ultra-low thermal conductivity. Part II: Temperature dependence of thermophysical properties. J. Mater. Sci..

[18] Miao J., Song X., Xu J., Xu J., Duan Z., Song Y., Gao L., Han Y., Ran X. (2025). Ultralight, Elastic, Thermally Insulating, and High-Temperature Resistant Al_2_O_3_–SiO_2_–B_2_O_3_ Nanofibrous Aerogels Prepared via the Direct Foaming Method. ACS Appl. Mater. Interfaces.

[19] Tao X., Tang L., Zhao Y., Zhao F., Xiang Z., He X., Wang M. (2026). Hierarchically structured aluminum borate whisker aerogel via sol–gel method and in-situ reaction. J. Adv. Ceram..

[20] Hu H., Wang J., Li C., Li L. (2024). Reinforcement Strategies to Improve the Mechanical Properties of Nanofibrous Aerogels: A Review. ACS Appl. Nano Mater..

[21] Lu D., Zhuang L., Zhang J., Su L., Niu M., Yang Y., Xu L., Guo P., Cai Z., Li M. (2023). Lightweight and Strong Ceramic Network with Exceptional Damage Tolerance. ACS Nano.

[22] Scheithauer U., Kerber F., Fuessel A., Holtzhausen S., Beckert W., Schwarzer E., Weingarten S., Michaelis A. (2019). Alternative Process Routes to Manufacture Porous Ceramics Opportunities and Challenges. Materials.

[23] Liang Y., Jin J., Yan J., Liu Q., Liu S., Wang X., Chen M., Qiu H. (2025). Preparation and Properties of Al_2_O_3f_/Al_2_O_3_-SiC Ceramic Matrix Composite. Adv. Ceram..

[24] Xu H., Li X., Tong Z., Zhang B., Ji H. (2022). Thermal Radiation Shielding and Mechanical Strengthening of Mullite Fiber/SiC Nanowire Aerogels Using In Situ Synthesized SiC Nanowires. Materials.

[25] Yuan Q., Gu Y., Cheacharoen R., Zhao J. (2026). Scalable Synthesis of Ultrathin Silica Nanosheets for Applications in Thermal-Management Aerogels and Flexible Composite Films. Rare Met..

[26] Fu J., Lian W., Deng Y., Fang Z., Cheng Q. (2025). Isotropic thermal insulating cuttlebone-inspired MXene aerogel. Natl. Sci. Rev..

[27] Zhu M., Li G., Gong W., Yan L., Zhang X. (2021). Calcium-Doped Boron Nitride Aerogel Enables Infrared Stealth at High Temperature Up to 1300 °C. Nano-Micro Lett..

[28] Ji C., Yan C., Wei Z., Yu T., Fan J., Li Y. (2024). Balancing thermal insulation and mechanical performance of anisotropic MXene foam enhanced by MOF hollow Framework. Chem. Eng. J..

[29] Hu C., Chen L., Lei Z., Li Y., Wang L., Yang Y., Zhao T., Li H. (2026). Tailoring the Microstructure and Mechanical Properties of Phenolic Aerogels with Graphene Oxide. Gels.

[30] Ji Q., Zhang L., Jiao X., Chen D. (2023). Alpha Al_2_O_3_ Nanosheet-Based Biphasic Aerogels with High-Temperature Resistance up to 1600 °C. ACS Appl. Mater. Interfaces.

[31] Xu X., Zhang Q., Hao M., Hu Y., Lin Z., Peng L., Wang T., Ren X., Wang C., Zhao Z. (2019). Double-negative-index ceramic aerogels for thermal superinsulation. Science.

[32] Hong S., Messing G. (1999). Anisotropic grain growth in boria-doped diphasic mullite gels. J. Eur. Ceram. Soc..

[33] Xiao Z., Wang W., Li X., Zhang L., Zhang T., Gao M., Kong L.B., Zhou K., Liu Y. (2021). Progress in fabrication and characterization of mullite whiskers. J. Micromech. Mol. Phys..

[34] Jiang J., Zhao W., Zhao L. (2024). Ultrarapid Gelation of Porous Ti_3_C_2_T_x_ MXene Monoliths Induced by Ionic Liquids. Nano Lett..

[35] Sergiienko S., Kovalevsky A., Luxa J., Mosina K., Wu B., Constantinescu G., Azadmanjiri J., Shcherban N., Diyuk O., Sofer Z. (2025). Engineering MXene/metal composites from MAX phase/metal–Al precursors for high-performance energy conversion and storage. RSC Adv..

[36] Wang X., Zhang Y., Zhi C., Wang X., Tang D., Xu Y., Weng Q., Jiang X., Mitome M., Golberg D. (2013). Three-dimensional strutted graphene grown by substrate-free sugar blowing for high-power-density supercapacitors. Nat. Commun..

[37] Guo Q., Zhang Y., Zhang H., Liu Y., Zhao Y., Qiu J., Dong G. (2017). 3D Foam Strutted Graphene Carbon Nitride with Highly Stable Optoelectronic Properties. Adv. Funct. Mater..

[38] Colombo P. (2006). Conventional and novel processing methods for cellular ceramics. Philos. Trans. R. Soc. A-Math. Phys. Eng. Sci..

[39] Shao G., Hanaor D., Shen X., Gurlo A. (2020). Freeze Casting: From Low-Dimensional Building Blocks to Aligned Porous Structures-A Review of Novel Materials, Methods, and Applications. Adv. Mater..

[40] Vakifahmetoglu C., Zeydanli D., Colombo P. (2016). Porous polymer derived ceramics. Mater. Sci. Eng. R. Rep..

[41] Wang Z., Guo A., Yan L., Zhang X., Ma J., Hou F., Liu J. (2025). Sugar blowing induced honeycomb porous mullite powders with low thermal conductivity and thermal stability. J. Alloys Compd..

[42] Wang Z., Guo A., Yan L., Zhang X., Ma J., Hou F., Liu J. (2026). Hierarchical Porous Mullite Fiber-Based Ceramics With Excellent Thermal Insulation and Mechanical Properties. J. Am. Ceram. Soc..

[43] Matuda T., Hoshino L., Ribeiro E., Tadini C. (2023). The influence of polydextrose on freeze-dried unripe acerola (Malpighia emarginata DC.) by the concept of a state diagram. J. Food Eng..

[44] Du Y., Peng K., Chen H., Su L., Niu M., Lu D., Wang H. (2025). SiC nanowire aerogel/NH_4_Cl composite as active-passive collaborative thermal protection material. J. Am. Ceram. Soc..

[45] Lv W., Zhang Q., Wang C., Wang T., Long J., Xu Y., Ma L. (2015). Interaction among bio-oil model components during oxidative degradation. Biomass Bioenerg..

[46] Xu H., Li D., Luo R., Zhao Y., Su H. (2025). Low-temperature synthesis of mullite and its structural evolution during mullitization. Ceram. Int..

[47] Krishnamoorthy K., Veerapandian M., Yun K., Kim S. (2013). The chemical and structural analysis of graphene oxide with different degrees of oxidation. Carbon.

[48] Karnis I., Krasanakis F., Sygellou L., Rissanou A., Karatasos K., Chrissopoulou K. (2024). Varying the degree of oxidation of graphite: Effect of oxidation time and oxidant mass. PCCP.

[49] Soria J., Sanz J., Sobrados I., Coronado J., Maira A., Hernández-Alonso M., Fresno F. (2007). FTIR and NMR Study of the Adsorbed Water on Nanocrystalline Anatase. Phys. Chem. C.

[50] Sabouni H., Jalilov A. (2026). Glucose-Derived Fluorescent Carbon Dots: Effect of Synthesis Temperature on Structural Evolution. ACS Omega.

[51] Pang Z., Chen Z., Li J., Liu D., Zhang G., Liu C., Du C., Zhou W. (2024). Advances in Inorganic Foam Materials Fabricated Via Blowing Strategy: A Comprehensive Review. ACS Nano.

[52] Liu K., Jin H., Huang L., Luo Y., Zhu Z., Dai S., Zhuang X., Wang Z., Huang L., Zhou J. (2022). Puffing ultrathin oxides with nonlayered structures. Sci. Adv..

[53] Qiu L., Liu J., Chang S., Wu Y., Li D. (2012). Biomimetic superelastic graphene-based cellular monoliths. Nat. Commun..

[54] Deville S. (2008). Freeze-Casting of Porous Ceramics: A Review of Current Achievements and Issues. Adv. Eng. Mater..

[55] Deville S., Saiz E., Tomsia A. (2007). Ice-templated porous alumina structures. Acta Mater..

[56] Mirabolghasemi A., Akbarzadeh A., Rodrigue D., Therriault D. (2019). Thermal conductivity of architected cellular metamaterials. Acta Mater..

[57] Kaviany M. (1995). Principles of Heat Transfer in Porous Media.

[58] Nield D.A., Bejan A. (2013). Convection in Porous Media.

[59] Wang K., Pera-Titus M. (2024). Microstructured gas-liquid-(solid) interfaces: A platform for sustainable synthesis of commodity chemicals. Sci. Adv..

[60] Zhan K., Chen Y., Xiong Z., Zhang Y., Ding S., Zhen F., Liu Z., Wei Q., Liu M., Sun B. (2024). Low thermal contact resistance boron nitride nanosheets composites enabled by interfacial arc-like phonon bridge. Nat. Commun..

[61] Olevsky E. (1998). Theory of sintering: From discrete to continuum. Mater. Sci. Eng. R-Rep..

[62] Wang Z., Feng P., Wang X., Geng P., Akhtar F., Zhang H. (2016). Fabrication and properties of freeze-cast mullite foams derived from coal-series kaolin. Ceram. Int..

[63] Yang J., Zhang X., Zhang B., Liu F., Li W., Jian J., Zhang S., Yang J. (2022). Mullite ceramic foams with tunable pores from dual-phase sol nanoparticle-stabilized foams. J. Eur. Ceram. Soc..

[64] Wan Y., Li X., Ma J. (2023). Mullite porous ceramics with high strength for high-temperature thermal insulation. J. Mater. Res. Technol..

[65] Wu Z., Xu T., Xu X., Dong X., Wang Z., Wu J., Yan L., Liu J., Guo A. (2024). Fabrication of mullite micro/nano fiber-based porous ceramic with excellent mechanical and thermal insulation properties. Ceram. Int..

[66] Lin L., Xu J., Wang H., Xia C., Shao Y., Feng X., Zhang P., Gao F. (2024). Near-net-size porous mullite ceramics with tuneable sintering shrinkage for thermal insulation applications. Ceram. Int..

