# Supplementary material for: Hierarchically Porous Mullite Ceramics Assembled from Ultrathin Nanosheets for High-Temperature Thermal Insulation
Source: Materials (Basel). 2026 Aug 13;19(16):3433. doi: 10.3390/ma19163433 (PMC13514568; doi:10.3390/ma19163433)
Supplement: Supplementary file 1 [file materials-19-03433-s001.zip › materials-4457711-supplementary.pdf]

## Supporting Information

# Hierarchically porous mullite ceramics assembled from ultrathin nanosheets for high-temperature thermal insulation

Zhongyan Wang [1], Anran Guo [1,*], Xueying Zhang [1], Jiaomei Ma [2], Jiachen Liu [1,*]

[1] School of Materials Science and Engineering, Key Lab of Advanced Ceramics and Machining Technology of Ministry of Education, Tianjin University, Tianjin 300072, China;

[2] Tianjin Cement Industry Design&Research Institute Co., LTD, Tianjin 300131, China;

Correspondence: jcliutju@tju.edu.cn (J. Liu), arguo@tju.edu.cn (A. Guo)

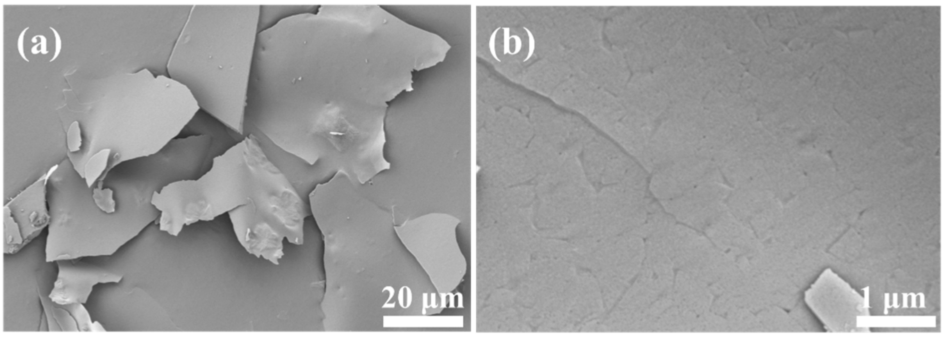


**Figure S1.** (a) SEM images of mullite nanosheets after milling: overall morphology, (b) High magnification of (a).

During the grinding and dispersion process, the lateral dimensions of the nanosheets were reduced (Figure S1a). As shown in Figure S1(b), large-area nanosheets can locally fragment, but this did not damage the unique two-dimensional morphology of the nanosheets.

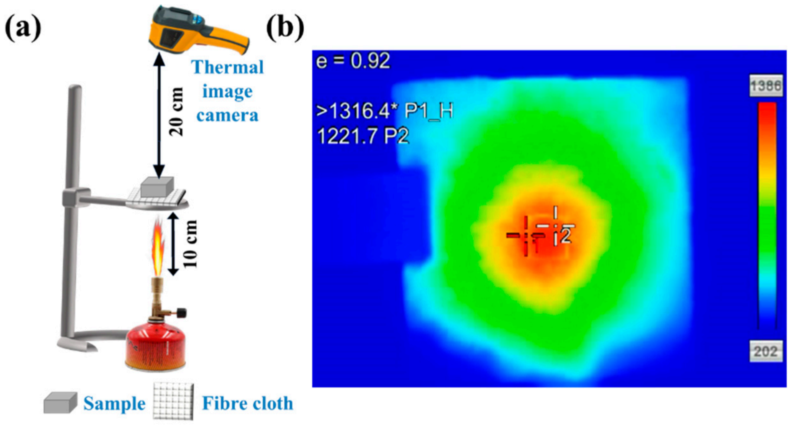


**Figure S2.** (a) Schematic illustration of the butane-flame thermal-insulation test, (b) infrared thermogram of the flame-facing surface measured under the same flame-exposure conditions.

As shown in Figure S2(a), the samples with the same dimensions were horizontally placed on the same fibre-cloth support. The distance between the burner nozzle and the sample surface was fixed at 10 cm, while the infrared thermal imaging camera was positioned approximately 20 cm from the monitored surface. The lateral surfaces of the samples were not additionally insulated during the flame test. The flame-facing surface temperature was independently measured by infrared thermography under the same flame-exposure conditions. As shown in Figure S2(b), with the emissivity set to 0.92, the temperature at the central heated region reached 1221.7 °C, while the local maximum temperature exceeded 1316.4 °C.

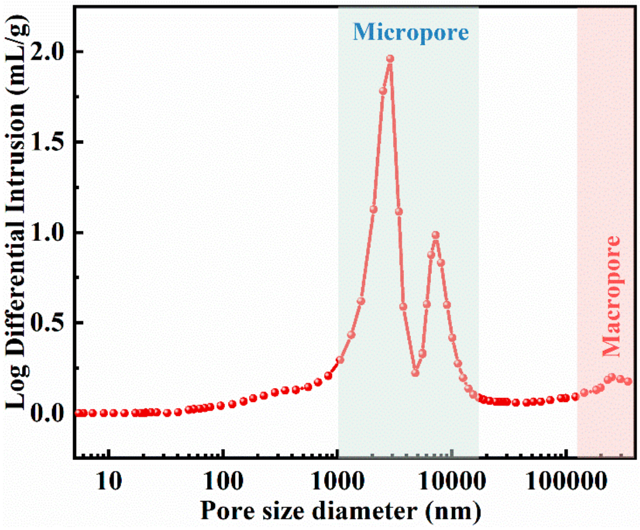


**Figure S3.** Pore structure distribution of the 20 wt.% porous ceramic sintered at 1200 °C.

To further characterize the hierarchical pore structure of the 20 wt.% sample, mercury intrusion porosimetry (MIP) was performed. As shown in Figure S3, two distinct peaks are observed at 2.9 μm and 6.6 μm, indicating the presence of micrometre-scale secondary pores. A weaker pore-size distribution is also observed at approximately 100-300 μm, indicating the presence of larger pores in the ceramic. This result is consistent with the SEM observations, indicating that both large pores and smaller secondary pores formed within interconnected nanosheet frameworks in the ceramic. This further demonstrates that the 20 wt.% sample had a hierarchical pore structure. The specific surface area is 5.007 $m^2/g$, indicating it had a large internal surface area and abundant solid-gas interfaces. These results further support the hierarchical pore structure observed by SEM and provide quantitative pore-structure information for understanding its thermal insulation behavior.

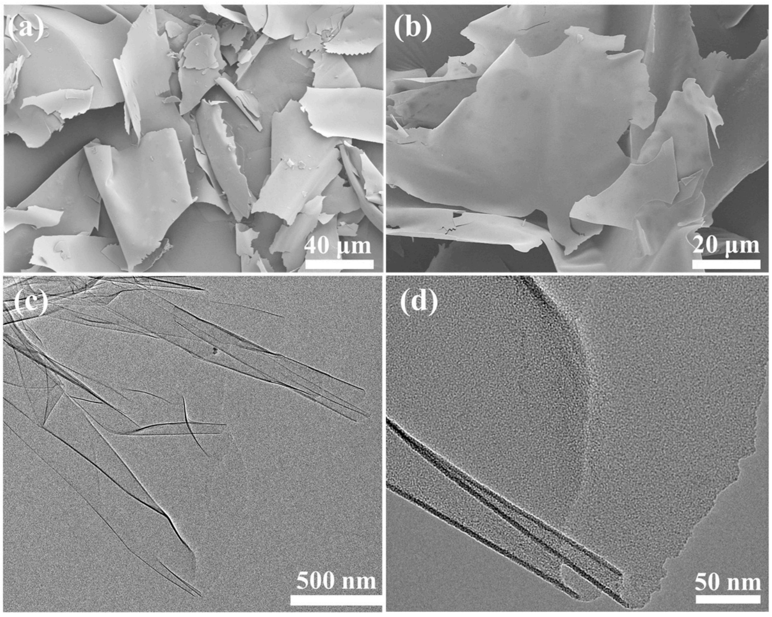


**Figure S4.** Morphological characterization of the ultrathin mullite nanosheets: (a, b) SEM image, (c, d) TEM image.

As shown in Figure S4(a-b), the SEM images reveal a typical sheet morphology, with individual nanosheets exhibiting relatively smooth surfaces and lateral dimensions of tens of micrometres. No obvious granular morphology is observed on the nanosheet surfaces. The TEM image in Figure S4(c) further shows extended electron-transparent sheets without obvious particulate aggregation, while the higher-magnification TEM image in Figure S4(d) confirms the continuous thin-sheet morphology. These observations demonstrate that the obtained nanosheets are continuous sheet-like structures rather than aggregates of discrete nanoparticles. The corresponding description of the formation mechanism has been revised accordingly in the manuscript, with the changes highlighted in red. The TEM images in Figure S4(c-d) further show thin, electron-transparent sheets with local folding and overlapping. In particular, the nanosheets remain continuous at higher magnification, without obvious boundaries or accumulation of discrete nanoparticles. These observations confirm that the products are continuous nanosheets rather than aggregates of nanoparticles.

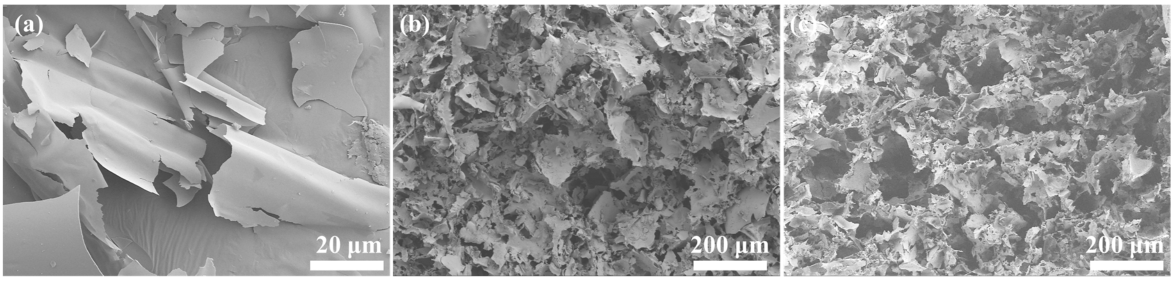

**Figure S5.** SEM images showing the structural evolution of mullite nanosheet-assembled porous ceramics during processing: (a) chemical blowing, (b) freeze-drying, (c) sintering.

As shown in Figure S5(a), the chemical blowing process was primarily employed for the synthesis of ultrathin mullite nanosheets rather than for directly constructing the pore structure of the porous ceramics. Then, these nanosheets were dispersed, gel cast, and freeze-dried to prepare porous ceramics. After dispersing the mullite nanosheets in the agarose/silica sol system, the agarose network provided temporary structural support to maintain the uniform distribution of nanosheets. During freezing, the growth of ice crystals acted as a pore-forming template, forcing the nanosheets to rearrange around the ice crystals. After sublimation, the space previously occupied by ice crystals transformed into interconnected macropores, while the stacking and overlapping of nanosheets generated secondary pores within the pore walls. As shown in Figure S5(b), the freeze-dried body exhibited a loosely assembled porous framework. However, because the agarose gel remained within the framework at this stage, some inter-sheet pores were partially occupied by the organic gel network, resulting in relatively incomplete pore connectivity. During the sintering process, agarose is gradually removed through thermal decomposition, thereby improving the connectivity of the pores. As shown in Figure S5(c), the connections between mullite nanosheets after sintering are further strengthened, forming a stable three-dimensional porous framework. Meanwhile, the large pores formed during the freezing process are still retained, resulting in an open and interconnected hierarchical pore structure for the sample
